# Diapause Induction and Termination in the Small Brown Planthopper, *Laodelphax striatellus* (Hemiptera: Delphacidae)

**DOI:** 10.1371/journal.pone.0107030

**Published:** 2014-09-04

**Authors:** LiuFeng Wang, KeJian Lin, Chao Chen, Shu Fu, FangSen Xue

**Affiliations:** 1 Institute of Entomology, Jiangxi Agricultural University, Nanchang, Jiangxi Province, China; 2 State Key Laboratory for Biology of Plant Diseases and Insect Pests, Institute of Plant Protection, Chinese Academy of Agricultural Sciences, Beijing, China; Natural Resources Canada, Canada

## Abstract

The small brown planthopper, *Laodelphax striatellus* (Fallén) enters the photoperiodic induction of diapause as 3rd or 4th instar nymphs. The photoperiodic response curves in this planthopper showed a typical long-day response type with a critical daylength of approximately 11 h at 25°C, 12 h at 22 and 20°C and 12.5 h at 18°C, and diapause induction was almost abrogated at 28°C. The third stage was the most sensitive stage to photoperiod. The photoperiodic response curve at 20°C showed a gradual decline in diapause incidence in ultra-long nights, and continuous darkness resulted in 100% development. The required number of days for a 50% response was distinctly different between the short- and long-night cycles, showing that the effect of one short night was equivalent to the effect of three long nights at 18°C. The rearing day length of 12 h evoked a weaker intensity of diapause than did 10 and 11 h. The duration of diapause was significantly longer under the short daylength of 11 h than it was under the long daylength of 15 h. The optimal temperature for diapause termination was 26 and 28°C. Chilling at 5°C for different times did not shorten the duration of diapause but significantly lengthened it when chilling period was included. In autumn, 50% of the nymphs that hatched from late September to mid-October entered diapause in response to temperatures below 20°C. The critical daylength in the field was between 12 h 10 min and 12 h 32 min (including twilight), which was nearly identical to the critical daylength of 12.5 h at 18°C. In spring, overwintering nymphs began to emerge in early March-late March when the mean daily temperature rose to 10°C or higher.

## Introduction

The small brown planthopper, *Laodelphax striatellus* (Fallén) (Hemiptera: Delphacidae) is one of the most serious and destructive pests of agriculture in temperate zones. The planthopper is a plant virus vector that attacks a wide range of economically important crops including rice, wheat, barley, corn and sugarcane causing significant damage [1]. *L. striatellus* is widely distributed in rice-producing regions throughout China and it has the potential to undertake long-distance migration [2], [3] and hibernate in temperate regions as 2–5th instar nymphs [4], [5]. This is in contrast with the economically important rice planthopper, *Nilaparvata lugens* and *Sogatella furcifera*, which are unable to overwinter in temperate regions in China and instead migrate into these regions every early summer.

Diapause is one of the primary mechanisms whereby insects synchronize their life cycles with local seasonal changes [6]. While undergoing dormancy, insects progress through a series of physiological phases, including diapause induction, maintenance and termination, post-diapause quiescence and post-diapause development. Each of these phases are strongly affected by photoperiod and temperature [7], [8]. Diapause in *L. striatellus* has been investigated under laboratory conditions in Japan. The nymphal diapause was induced by short daylengths at low temperatures during the nymphal development and short photoperiod maintained nymphal diapause of *L. striatellus* [9]–[11]. However, diapause in *L. striatellus* has not been reported in China. For a better understanding of insect life cycles, a detailed understanding of diapause in the planthopper would be desirable because such information is helpful in improving the prediction and management of this pest.

In the present study, diapause induction and termination of the small brown planthopper, *L. striatellus* were systematically investigated under laboratory and field conditions. The purpose of our experiments is to reveal the role of photoperiod and temperature in diapause induction and termination in this planthopper and how the planthopper decides to initiate and terminate diapause under field conditions of changing photoperiod and temperature.

## Materials and Methods

### Ethics Statement

Because the small brown planthopper *L. striatellus* is a serious and destructive pest of agriculture in the temperate zone in China, no permits were required for collecting the insect and performing the experiments. All experiments were carried out at the Institute of Entomology, Jiangxi Agricultural University, Nanchang, Jiangxi Province (28°46′ N, 115°49′ E).

### Experimental Materials and Insect Rearing Conditions

The original colony of *L. striatellus* was collected from the rice fields in the suburbs of Nanchang (28.8° N, 115.9° E), Jaingxi Province. Under local conditions the planthopper exhibits mixed voltinism from four to seven generations per year due to the differences in the diapause intensity of overwintering nymphs and the long ovipositional period of females and overwinters as 1–5 instar nymphs [12]. In the present study, nymphs in the overwintered and the first generations were raised with stems of American sloughgrass, *Beckmannia syzigache*; nymphs in the other generations were raised with rice stems. The planthoppers were reared at 25°C under LD 15∶9 (a diapause-averting photoperiod) for several generations before use.

Approximately 50 newly hatched nymphs were put in a glass tube (length: 180 mm; diameter: 32 mm) with stems of American sloughgrass or rice stems, and exposed to different photoperiods and temperatures to observe diapause induction. In the diapause termination experiments, diapausing nymphs were placed in glass tubes with rice stems and they were exposed to different photoperiods and temperatures to observe diapause termination.

All laboratory experiments were performed in illuminated incubators (LRH-250-GS, Guangdong Medical Appliances Plant, Guangdong, China) equipped with four fluorescent 30 W tubes with an irradiance of approximately 1.97 W m^−2^ and the variation in the temperatures was ±1°C.

### Diapause Induction Experiments

#### Photoperiodic Response

The photoperiodic responses for diapause induction in *L. striatellus* were experimentally determined under various photoperiods at constant temperatures of 18, 20, 22 and 25°C. The influence of unnatural photoperiods (including continuous darkness (DD) and continuous light (LL)) on diapause induction was also examined at 20°C. Diapausing individuals were easy to identify because nymphs entered diapause in 3–4th instars and showed a distinct developmental delay. Thus, the incidence of diapause was determined based on the proportion of nymphs that were still in 3–4th instars within one week after comparable control cultures had completed emergence because the nymphs has stopped development and were almost not feeding during this period. There were 39–129 individuals in each of three replicates for most treatments; a few treatments had two or more than three replicates.

### Required Day Number

The required day number (RDN), i.e., the number of light-dark cycles needed to raise the proportion of diapause in a population to 50% [13], was determined by transferring nymphs from a long photoperiod (LD 15∶9, a diapause-averting photoperiod) to a short photoperiod (LD 11∶13, a diapause-inducing photoperiod) at different times after hatching at 18°C or vice versa. There were 46–198 individuals in each of at least three replicates for all of the treatments.

### Detection of Photosensitivity

To determine the greatest sensitivity to diapause-inducing photoperiodic cues, an experiment was performed as described by Spieth [14] involving the periodic interruption of diapause-inducing conditions. Because all nymphs enter diapause at 18°C under the short daylength of LD 11∶13 and because five days comprise just one instar time, nymphs reared under a photoperiodic background of LD 11∶13 were interrupted by five short nights of LD 15∶9 at various nymphal stages at 18°C or vice versa. There were two or three replicates for each treatment.

### The Incidence of Diapause under Field Conditions

To understand how the planthoppers decide to diapause under field conditions of changing photoperiod and temperature, nymphs from the autumn generation that hatched at different times from mid-September to mid-November in 2011–2013 were collected daily or every other day then approximately 50 newly hatched nymphs were put in a glass tube with rice stems. There were one to fifteen replicates for each treatment, depending on the hatching amount each day. The nymphs were allowed to develop under outdoor conditions to observe the time course of diapause induction. The incidence of diapause was recorded until all nymphs entered diapause. The total number observed was 7896 nymphs in 2011, 4392 nymphs in 2012 and 6956 nymphs in 2013.

### Diapause Termination Experiments

#### Effect of Diapause-inducing Photoperiod and Temperature on Diapause Intensity

The effect of pre-diapause photoperiod and temperature on diapause intensity (duration) was evaluated by rearing newly hatched nymphs under the diapause-inducing daylengths of 10, 11 and 12 h at the constant temperatures of 18, 20 and 22°C until diapause determination. Diapausing nymphs induced under daylengths of 10, 11 and 12 h at 18, 20 and 22°C were transferred to LD 15∶9 and 25°C to test diapause development. The emerged adults were recorded every day until all of the diapausing nymphs emerged.

#### Effect of Diapause-terminating Photoperiod and Temperature on Diapause Intensity

Diapausing nymphs induced under LD 11∶13 at 18°C were divided into two groups. One group was incubated under a long photoperiod of LD 15∶9 and a short photoperiod of LD 11∶13 at 18°C to test the effect of photoperiod on diapause maintenance and termination. The other group was incubated under LD 15∶9 at different temperatures (18, 20, 22, 24, 26, 28, 30 and 32°C) to examine the effect of temperature on diapause maintenance and termination. The emerged adults were recorded every day until all of the diapausing nymphs emerged.

#### Effect of Chilling on Diapause Termination

To investigate the effect of chilling on diapause development, diapausing nymphs induced under LD 11∶13 at 18°C were placed at 5°C for different lengths of time (ranging from 20 to 80 days) in continuous darkness. After chilling, the nymphs were transferred to LD 15∶9 and 25°C to terminate diapause. The emerged adults were recorded every day until all of the diapausing nymphs emerged.

#### Diapause Termination under Field Conditions

The naturally diapausing nymphs that hatched at different times from mid-September to mid-November in 2011 and 2012 were kept under outdoor conditions to observe adult emergence in the next spring. The emerged adults were recorded every day until all overwintering nymphs emerged.

### Statistical Analyses

Statistical analyses were conducted using SPSS 19.0 (IBM Inc.). The effects of photoperiod (from 10 h to 16 h), temperature and their interactions on the induction of diapause were tested using a General Linear Model (GLM). The influence of diapause-inducing temperature and photoperiod, diapause-terminating temperature and the chilling period on the duration of diapause were tested using Kruskal–Wallis tests following non-parametric tests. The influence of diapause-terminating photoperiod on the duration of diapause was tested by independent-samples *t* test. Differences are considered significant if *P*<0.05.

## Results

### Photoperiodic Responses for Diapause Induction

**Figure 1:**
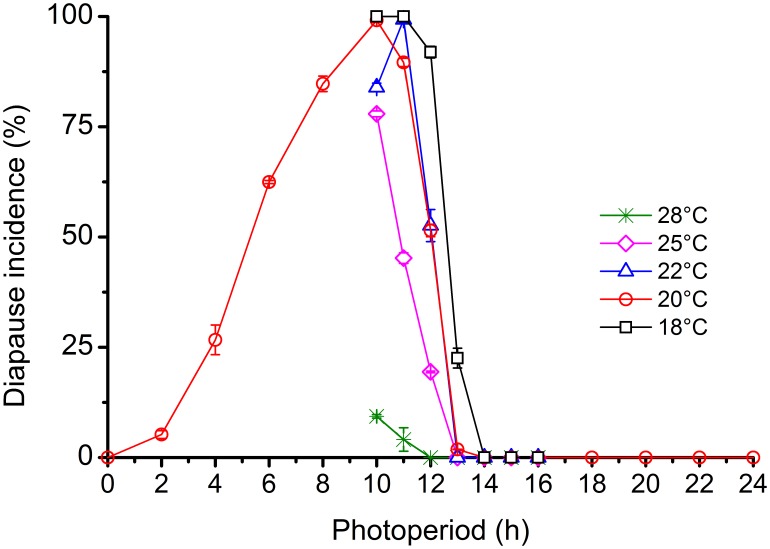
Photoperiodic response curves for diapause induction in *L. striatellus* at constant temperatures of 18, 20, 22 and 25°C. *N* = 79–303 for each point.

Photoperiodic response curves for diapause induction in *L. striatellus* at different temperatures are shown in Fig. 1. The photoperiodic response curves showed a typical long-day response type with a critical daylength of approx. 11 h at 25°C, 12 h at 22 and 20°C and 12.5 h at 18°C. The long daylengths of 13–16 h induced 100% development without diapause at 22 and 25°C and 77–100% development at 18 and 20°C. However, the high temperature of 28°C nearly abrogated the diapause-inducing effects of short daylengths; more than 90% individuals developed without diapause under short daylengths. The photoperiod, temperature and their interactions all have a significantly influence on the induction of diapause (Temperature effect: F_4,105_ = 1064.3, P = 0.000; Photoperiod effect: F_6,105_ = 3441.8, P = 0.000; Temperature × Photoperiod interactions: F_24,105_ = 208.5, P = 0.000). The photoperiodic response curve at 20°C showed a gradual decline in diapause incidence in ultra-long nights (from 16 h nightlength to 22 h nightlength), and DD resulted in 100% development.

### Required Day Number

**Figure 2:**
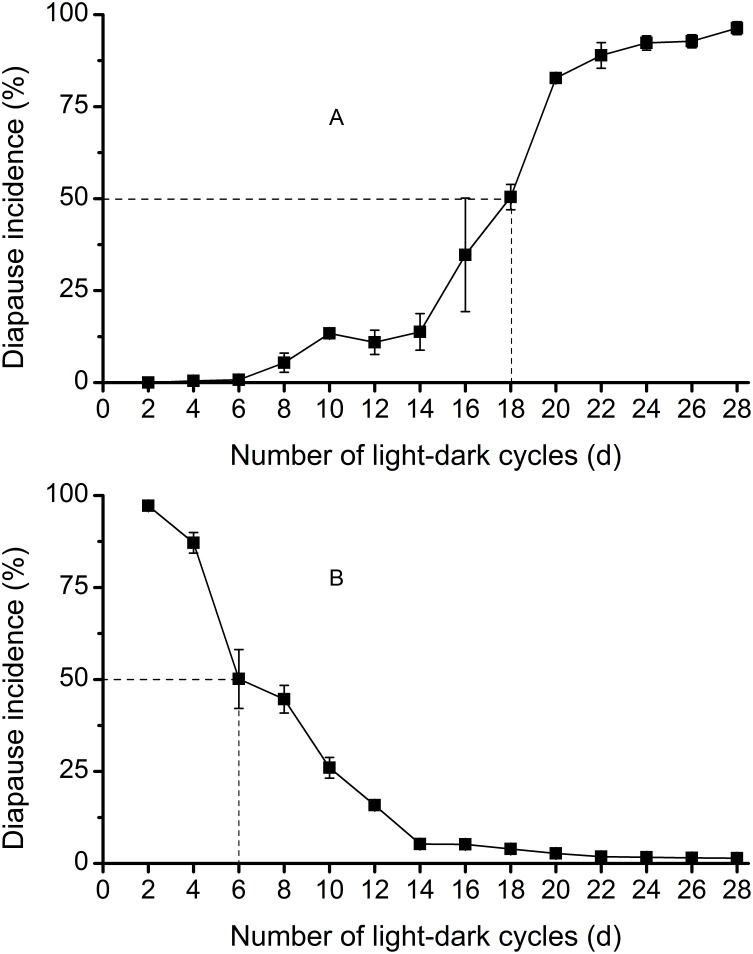
Incidence of diapause in *L. striatellus* at 18°C: (A) when nymphs were exposed to different numbers of long-night cycles (LD11∶13) and then moved to short-night cycles (LD 15∶9); (B) when nymphs were exposed to different numbers of short-night cycles (LD 15∶9) and then moved to long-night cycles (LD 11∶13). N = 137–352 for each point.

The RDN for 50% response was distinctly different between short- and long-night cycles at 18°C. It was 18 days for long-night cycles (Fig. 2A) and 6 days for short-night cycles (Fig. 2B), indicating that the effect of one short-night was equivalent to the effect of three long-nights.

### The Most Sensitive Stage to Photoperiod

**Figure 3:**
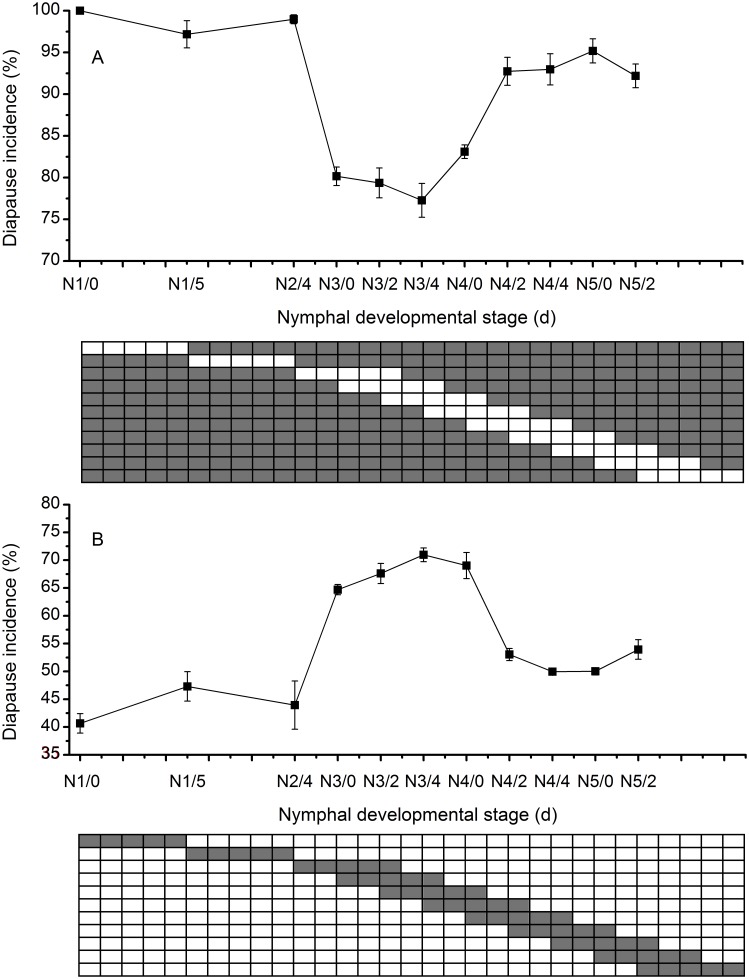
Photosensitivity of diapause during nymphal development at 18°C in *L. striatellus*: (A) when a background period of LD 11∶13 was interrupted by five long photoperiods of LD 15∶9; (B) when a background period of LD 15∶9 was interrupted by five short photoperiods of LD 11∶13. White bars represent the long photoperiod (LD15∶9), and gray bars represent the short photoperiod (LD11∶13). N3/4 means fourth day of third instar. *N* = 60–185 for each point.

When the diapause-inducing photoperiod of LD 11∶13 was interrupted by five long daylengths at 18°C, the most effective diapause inhibition occurred between the N3/0 (just entered third instar) and N3/4 (the fourth day of third instar) stages (Fig. 3A). Similarly, the diapause-inducing effects were also higher between the N3/0 and N3/4 stages when the diapause-inhibiting photoperiod of LD 15∶9 was interrupted by five short daylengths (Fig. 3B). The results suggest that the 3rd instar nymph is most sensitive to photoperiod in *L. striatellus*.

### The Time Course of Diapause Induction under Field Conditions

**Figure 4:**
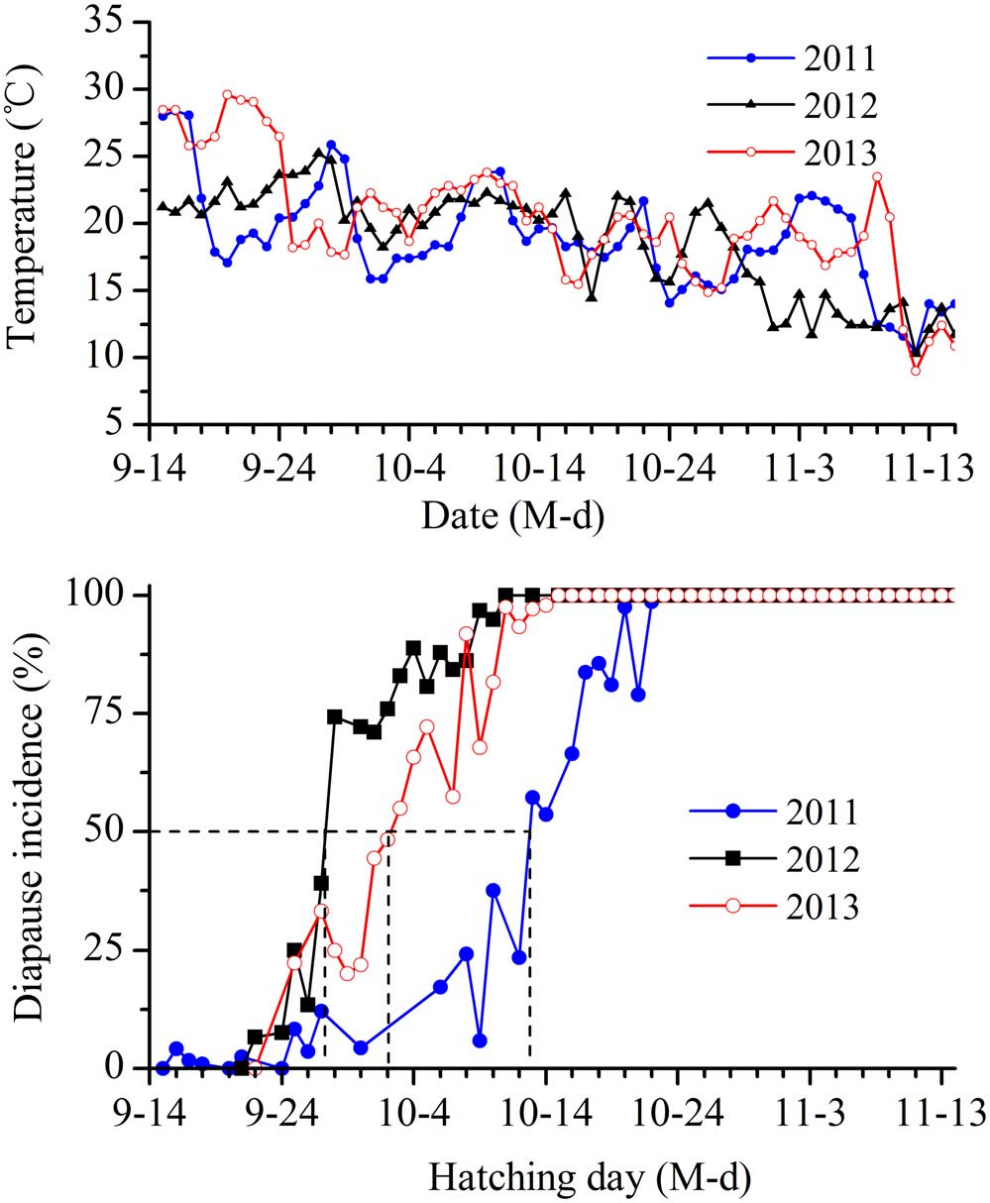
Incidence of nymphal diapause in *L. striatellus* which hatched on different dates under field conditions. *N* = 59–856 for each point.

Fig. 4 shows the time course of diapause induction under field conditions for three years. Winter diapause had already occurred in some individuals that hatched on 16 September in 2011, 22 September in 2012 and 25 September in 2013. The proportion of diapausing individuals increased with time, and 50% nymphs initiated diapause on 13 October in 2011, 28 September in 2012 and 2 October in 2013. As shown above, the 3rd instar larva was the stage that was most sensitive to photoperiod. The nymphs required approximately 8 days to finish the first and second instars after hatching in late September or mid-October. Therefore, the photosensitive stage started 8 days after hatching, i.e., on 21 October in 2011, 6 October in 2012, and 10 October in 2013. By consulting the astronomical yearbooks [15], the daylength in the Nanchang region between October 6 and October 21 was between 12 h 10 min and 12 h 32 min (including twilight), which was the critical daylength for diapause induction in the field. Nymphs that hatched after mid-October all entered diapause when the mean daily temperature experienced by nymphs was lower than 20°C.

### Effect of Diapause-inducing Photoperiod and Temperature on Diapause Intensity

**Figure 5:**
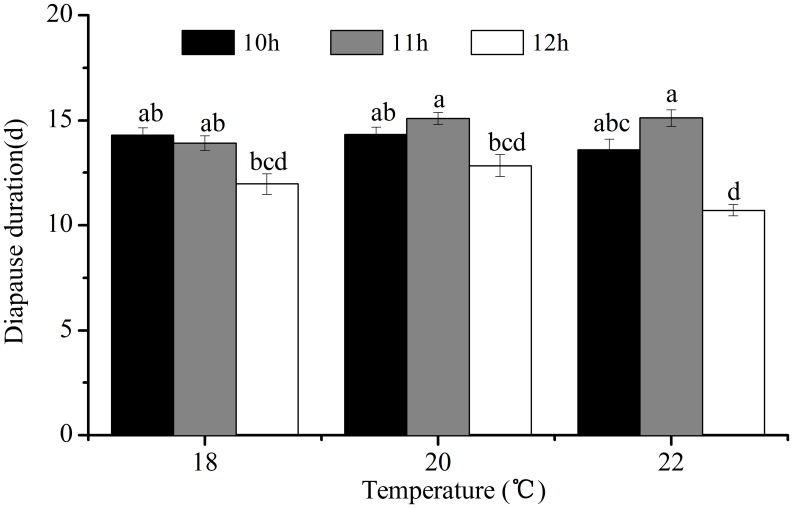
Diapause duration under LD 15∶8 at 25°C in *L. striatellus*. Diapause was induced by the short daylengths of 10, 11 and 12 h at 18, 20 and 22°C. *N* = 26–48 for each treatment.

The diapause-inducing photoperiod had a significant effect on diapause intensity (Fig. 5). The duration of diapause induced by a photoperiod of LD 12∶12 was significantly shorter than that of LD 11∶13 at 20°C and shorter than those of LD 10∶14 and LD 11∶13 at 22°C (*χ* ^2^ = 80.39, *d.f.* = 8, *P*<0.05), whereas diapause-inducing temperature had no significant effect on diapause intensity at different photoperiods (*P*>0.05).

## Effect of Diapause-terminating Photoperiod and Temperature on Diapause Intensity

**Figure 6:**
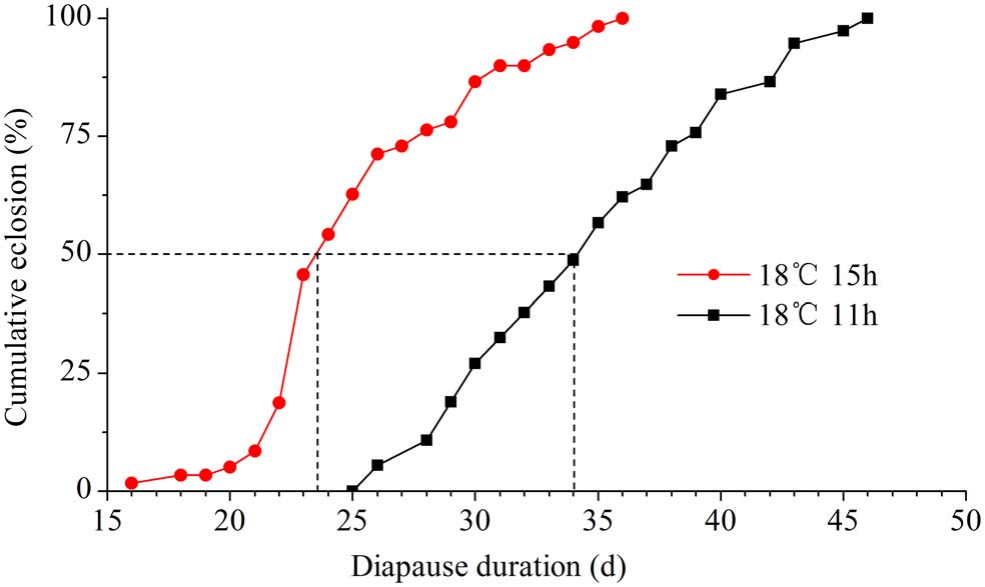
The duration of diapause of *L. striatellus* under the daylengths of 11 and 15 h at 18°C. Diapause was induced under LD 11∶13 at 18°C. *N* = 42–79 for each point.

Diapause ended even at a short daylength of LD 11∶13; however, the duration of diapause was significantly longer under the short daylength of LD 11∶13 (35 days) than it was under the long daylengths of LD 15∶9 (26 days) (*t* = 8.97, *d.f.* = 63, *P*<0.01) (Fig. 6), indicating that long photoperiods can accelerate diapause development.

**Figure 7:**
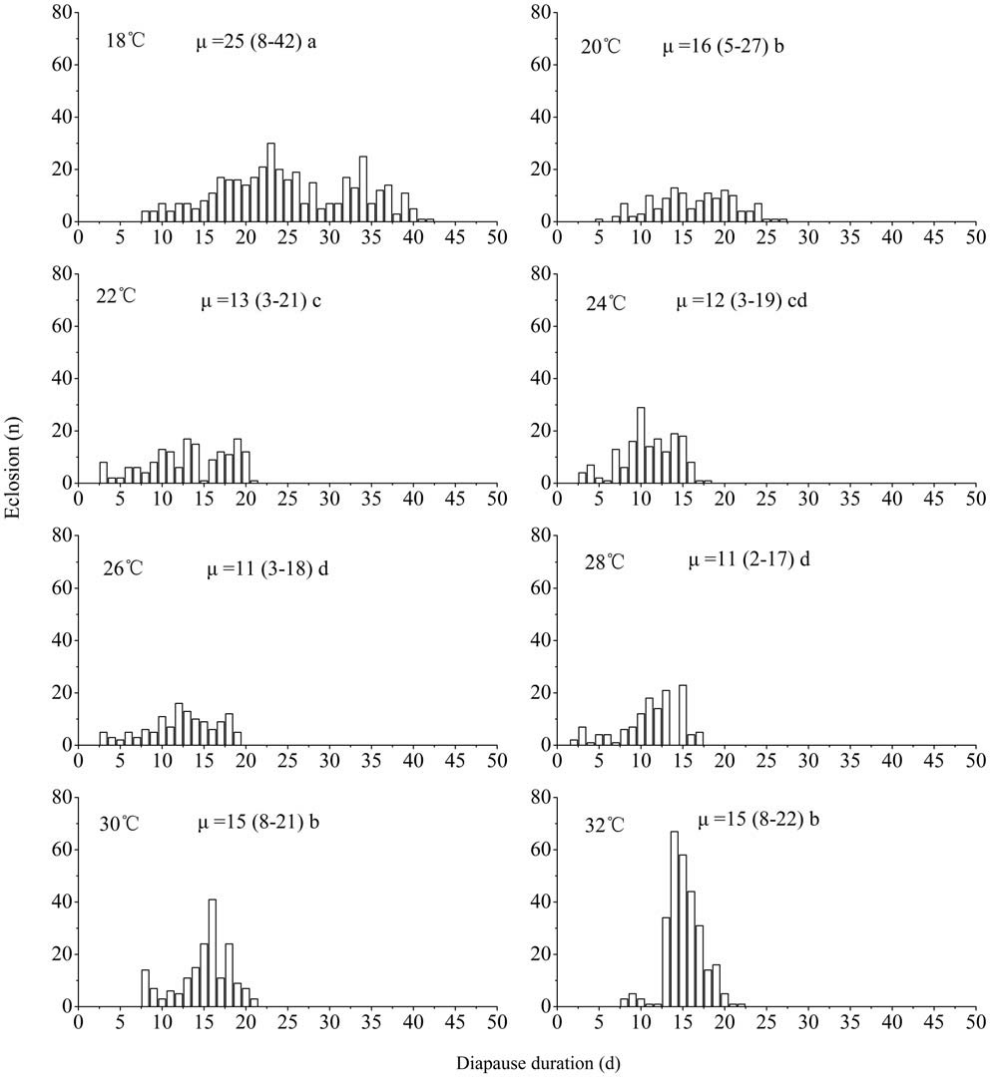
Diapause termination under LD 15∶8 at different temperatures in *L. striatellus*. Diapause was induced under LD 11∶13 at 18°C. *N* = 127–393 for each point.

In addition, diapause-terminating temperature significantly influenced the diapause intensity (*χ^2^* = 711.42, *d.f.* = 7, *P*<0.05) (Fig. 7). The duration of diapause was gradually shortened from 25 days at 18 to 11 days at 28°C. Furthermore, the eclosion was less synchronous at 18°C than it was at other temperatures which resulted in changes in the mean, but a substantial proportion of the individuals still eclosed between 10–20 days. However, at the high temperatures of 30 and 32°C, the duration of diapause was significantly longer than the durations at the temperatures of 22, 24, 26 and 28°C. This result indicates that the optimal temperature for diapause termination is in the 26–28°C range.

## Effect of Chilling on Diapause Termination

**Figure 8:**
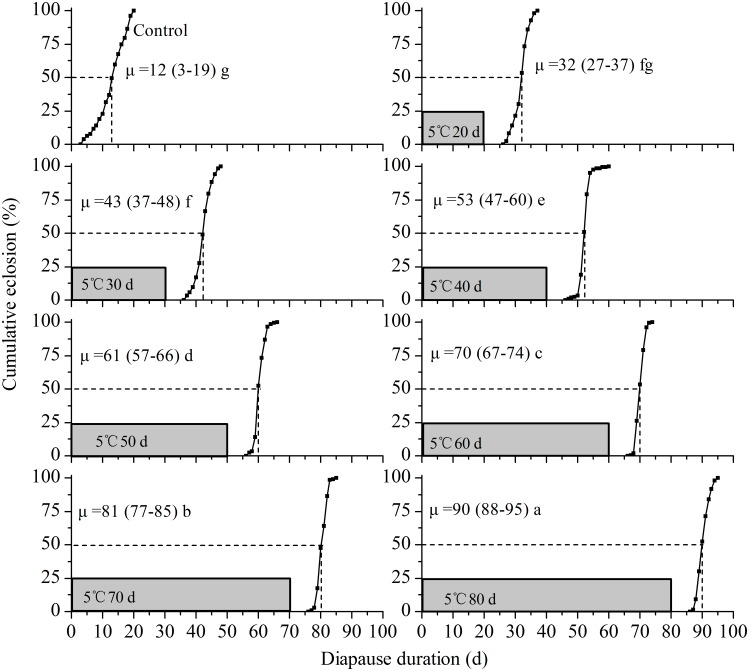
Effect of chilling on diapause termination in *L. striatellus*. Diapausing nymphs induced under LD 11∶13 at 18°C were transferred to 5°C DD for 0, 20, 30, 40, 50, 60, 70 and 80 days, and subsequently transferred to LD 15∶9 and 25°C. N = 122–200 for each treatment.

Fig. 8 shows the cumulative eclosion rate of diapausing nymphs under LD 15∶9 at 25°C after chilling at 5°C for 20, 30, 40, 50, 60, 70 and 80 days. The eclosion time was significantly postponed with increasing chilling duration if the time spent under chilling was considered as part of the duration of diapause development (*χ^2^* = 1245.57, *d.f.* = 7, *P*<0.01), indicating that chilling at 5°C did not shorten the duration of diapause but lengthened it. In fact, diapausing nymphs without chilling (control) all emerged within 16 days when they were transferred to LD 15∶9 at 25°C.

## Time Course of Diapause Termination under Field Conditions

**Figure 9:**
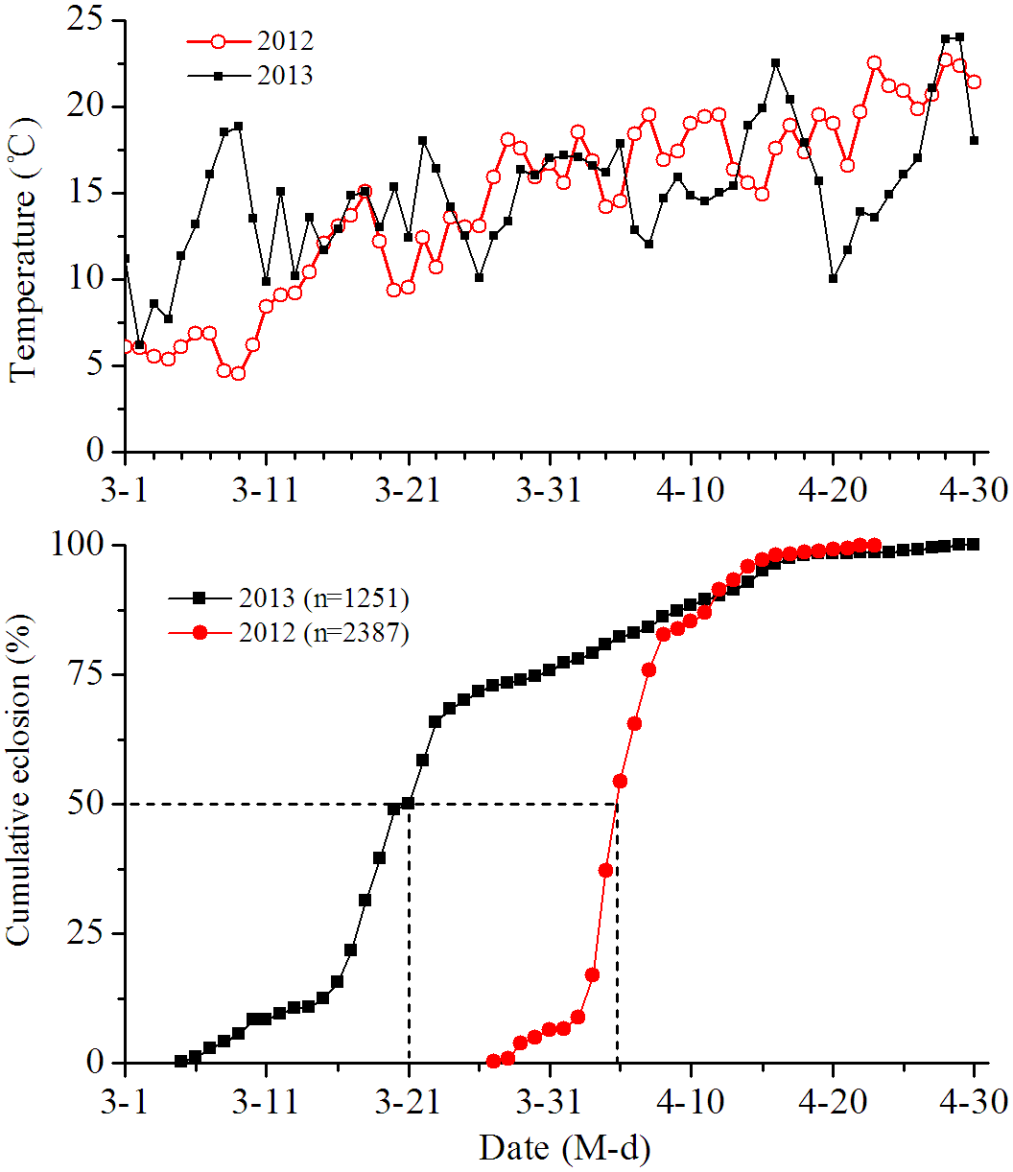
Adult eclosion of overwintering generation of *L. striatellus* under field conditions in spring.

The cumulative eclosion rate of the naturally overwintering nymphs is presented in Fig. 9. A few overwintering nymphs began to emerge on 27 March 2012 and 5 March in 2013 when the mean daily temperature rose to 10°C or higher. A proportion of 50% eclosion occurred on 4 April 2012 and on 20 March 2013, showing a 15 day difference. This is most likely because the mean daily temperature in March was 2.8°C lower in 2012 (10.6°C) than it was 2013 (13.4°C). It suggests that the temperatures in March determine the rate of post-diapause development in the planthopper.

## Discussion

The photoperiodic response for diapause induction in *L. striatellus* highly depended on temperature. The critical daylength decreased with an increase in temperature from 18 to 25°C (11 h at 25°C, 12 h at 22 and 20°C and 12.5 h at 18°C; Fig. 1). The critical daylength could not be determined at the high temperature of 28°C because this temperature significantly weakened and even completely inhibited the photoperiodic induction of diapause (more than 90% of the individuals developed without diapause under short daylengths) However, it is important to note that the critical daylengths of 11 h at 25°C and 12 h at 22 and 20°C do not exist in the Nanchang region. In Nanchang, the longest period of daylight in a year is approximately 14 h 56 min (including twilight), the shortest is 11 h 9 min. Therefore, an 11 h daylength occurs in late winter and a 12 h daylength occurs at the end of October. Apparently, the critical daylengths at 20, 22 and 25°C were not suitable to analyze the incidence of diapause in the field. Only the critical daylength of 12.5 h at 18°C was related to diapause induction in the field because this result was consistent with the photoperiod experienced by nymphs entering diapause in the field (12 h 10 min-12 h 32 min; Fig. 4). Our results indicate that the nymphs entered winter diapause in response to short daylengths and low temperatures during autumn, which is consistent with the result reported by Kisimoto [9].

The photoperiodic response curve at 20°C in *L. striatellus* showed a gradual decline in diapause incidence during ultra-long nights (from 16 h nightlength to 22 h nightlength) and DD (Fig. 1), suggesting that different long nights have different inductive effects. This phenomenon has been found in many long-day insect species, such as the large white butterfly, *Pieris brassicae* [16], the Indian meal moth, *Plodia interpunctella* [17], the linden bug, *Pyrrhocoris apterus* [18], the fly, *Chymomyza costata* [19], the spider mite, *Tetranychus urticae* [20], the pine caterpillars species, *Dendrolimus punctatus* and *D. tabulaeformis* [21], [22], the endoparasitoid wasp *Microplitis mediator* [23], and the rice stem borer *Chilo suppressalis* [24]. That ultra-long nights and DD result in a decline in diapause incidence presumably reflects the absence of selective pressure, but it may have a physiological significance when one attempts to determine the mechanism of time measurement [25].

One basic concept concerning photoperiodic responses in insects is the RDN [13]. It has been shown that a greater number of exposure days is required for the induction of diapause than are required for its termination [8]. This has been indicated in species as diverse as the mosquito *Aedes atropalpus*, the aphid *Megoura viciae*, the cabbage white butterfly *Pieris rapae*, the European grapevine moth *Lobesia botrana*, the Asian swallowtail *Papilio xuthus* [8], and the zygaenid moth *Pseudopidorus fasciata* [26] and *D. punctatus* [21]. The transfer experiments in *L. striatellus* (Fig. 2) showed that the RDN for short nights was three times less than for that long nights at 18°C (cf. 6 days vs. 18 days), indicating that short nights are photoperiodically more potent.

The intensity of diapause dose not only vary between species but also among individuals of the same species depending on how long they have been exposed to diapause-inducing and terminating conditions [15]. According to the present findings, the intensity of diapause in *L. striatellus* nymphs was affected by diapause-inducing photoperiods. The duration of diapause induced by a photoperiod of LD 12∶12 was significantly shorter than those by LD 10∶14 and LD 11∶13 at 20 and 22°C (Fig. 5). Similar results have been reported for a number of insects, such as the fruit flies, *Drosophila auraria*, *D. subauraria*, *D. triauraria*, the bean bug, *Riptortus clavatus*, the Mediterranean tiger moth, *Cymbalophora pudica* and the cotton bollworm, *Helicoverpa armigera* [27]–[31], where photoperiods with longer scotophases induced more intense diapause compared with shorter scotophases. Our results further showed that the duration of diapause in *L. striatellus* was significantly affected by the photoperiod during diapause. By transferring diapausing nymphs to a short photoperiod of LD 11∶13 and a long photoperiod of LD 15∶9 combined with 18°C, diapause was terminated significantly faster at LD 15∶9 than it was at LD 11∶13 (Fig. 6), suggesting that short daylengths may play a role in maintaining winter diapause. Similar results have also been reported for the mulberry tiger moth *Spilarctia imparilis* [32], the lacewing *Chrysopa downesi* [33] and the cabbage butterfly *Pieris melete*, where long photoperiods during diapause significantly accelerated winter diapause development.

Increasing evidence has shown that chilling is not a prerequisite for the completion of hibernation diapause in many insect species and diapause completion progresses well at intermediate or high temperatures in some insects [24], [34]–[39]. Our data revealed that the diapause could be terminated without exposure to chilling in *L. striatellus*. The rate of diapause completion was positively related to the temperature increase between 18 and 28°C. However, diapause development was delayed when the temperature rose to 30 and 32°C (Fig. 7). This result suggests that the optimal temperature for diapause development is 26 and 28°C in *L. striatellus*. The chilling experiments in Fig. 8 showed that the eclosion time of diapausing nymphs was significantly postponed with an increase in chilling time if the time spent under chilling was considered as part of the duration of diapause development, indicating that chilling at 5°C did not shorten the duration of diapause but lengthened it. Our results suggest that low temperatures during winter may serve primarily to maintain nymphal diapause and prevent the resumption of post-diapause morphogenesis, which in turn synchronizes the adult emergence of the overwintering generation with the availability of host plants [40].

A few studies have examined diapause induction and termination under field conditions of changing photoperiod and temperature [24], [41]. In the present study, we systematically investigated the time course of diapause induction and termination of *L. striatellus* under field conditions. Our results reveal that winter diapause had already occurred in some individuals that hatched in mid-September or late September; 100% of the nymphs that hatched after mid-October entered diapause when the mean daily temperature experienced by nymphs decreased to 20°C or lower (Fig. 4). This result suggests that the important cue for the initiation of winter diapause depends primarily on temperature, i.e., the temperatures between mid-September and mid-October. Observations of diapause termination under field conditions reveal that a few diapausing nymphs initiated their eclosion on 5 March or 25 March when the mean daily temperature rose to 10°C or higher (Fig. 9), suggesting that an early or late termination of winter diapause primarily depends on the temperatures in March. Therefore, these data indicate that temperature was strongly correlated with the induction and termination in *L. striatellus*. Combining our results with the climatic data from the locality, we can predict the time of diapause initiation in autumn and adult emergence in spring for this insect.

Furthermore, our results emphasize the importance of understanding how insects decide to initiate and terminate diapause under field conditions of changing photoperiod and temperature. As long as we do not understand this, we can never be certain how the findings from the laboratory relate to conditions in the field.
